# Data-Driven Design and Additive Manufacturing of Patient-Specific Lattice Titanium Scaffolds for Mandibular Bone Reconstruction

**DOI:** 10.3390/jfb16090350

**Published:** 2025-09-18

**Authors:** Nail Beisekenov, Bagdat Azamatov, Marzhan Sadenova, Dmitriy Dogadkin, Daniyar Kaliyev, Sergey Rudenko, Boris Syrnev

**Affiliations:** 1 Department of Ecology and Conservation Biology, Texas A&M University, College Station, TX 77843, USA; beisekenovnail@tamu.edu; 2 Graduate School of Science and Technology, Niigata University, Niigata 950-2181, Japan; 3 Smart Engineering Competence Centre, D. Serikbayev East Kazakhstan Technical University, 19 Serikbayev Street, Ust-Kamenogorsk 070010, Kazakhstan; bazamatov@edu.ektu.kz (B.A.); ddogadkin@edu.ektu.kz (D.D.); dkaliyev@edu.ektu.kz (D.K.); srudenko@edu.ektu.kz (S.R.); 4 Center of Excellence “VERITAS”, D. Serikbayev East Kazakhstan Technical University, Ust-Kamenogorsk 070004, Kazakhstan; bsyrnev@edu.ektu.kz

**Keywords:** patient-specific implant design, finite-element modelling (FEM), artificial neural network (ANN), Bayesian network (BN), additive manufacturing (AM), direct metal laser sintering (DMLS), material property prediction, lattice architecture

## Abstract

The reconstruction of segmental bone defects requires patient-specific scaffolds that combine mechanical safety, biological functionality, and rapid manufacturing. We converted CT-derived mandibular geometry into a functionally graded Ti-6Al-4V lattice and optimised porosity, screw layout, and strut thickness through a cyber-physical loop that joins high-fidelity FEM, millisecond ANN, and a BN for uncertainty quantification. Fifteen candidate scaffolds were fabricated by direct metal laser sintering and hot isostatic pressing and were mechanically tested. FEM predicted stress and stiffness with 98% accuracy; the ANN reproduced these outputs with 94% fidelity while evaluating 10,000 designs in real time, and the BN limited failure probability to <3% under worst-case loads. The selected 55–65% porosity design reduced titanium use by 15%, shortened development time by 25% and raised multi-objective optimisation efficiency by 20% relative to a solid-plate baseline, while resisting a 600 N bite with a peak von Mises stress of 225 MPa and micromotion < 150 µm. Integrating physics-based simulation, AI speed, and probabilistic rigour yields a validated, additively manufactured scaffold that meets surgical timelines and biomechanical requirements, offering a transferable blueprint for functional scaffolds in bone and joint surgery.

## 1. Introduction

Personalised orthopaedic and cranio-maxillofacial surgery increasingly depends on implants whose macro-geometry, lattice architecture, and surface chemistry are tuned to a single patient’s anatomy, load path, and healing biology. Delivering such bespoke devices in routine practice demands digital pipelines able to (i) capture inter-patient variability without prohibitive computation, (ii) predict long-term, multi-physics performance across high-dimensional design spaces, and (iii) quantify the uncertainty stemming from both biological inputs and additive-manufacturing (AM) process scatter.

Finite-element modelling (FEM) underpins most current pipelines, supporting analyses of stress shielding in hip stems, optimisation of stent struts, and micromotion studies at the bone–implant interface [1,2]. Accurate FEM prediction of stress shielding has been shown to extend the life of porous acetabular cups [2,3,4], and similar frameworks guide patient-matched cranio-maxillofacial and dental reconstructions reconstructed from CT data [3,4,5]. Nevertheless, FEM alone becomes impractical when thousands of permutations in anatomy, lattice gradation, and microstructure must be interrogated simultaneously [6,7,8,9].

Artificial neural networks (ANNs) mitigate this bottleneck by learning non-linear relationships and returning millisecond-scale surrogates [10,11,12,13,14]. ANN models have inferred the elastic modulus and yield strength of laser-powder-bed-fused Ti-6Al-4V from scan parameters [15,16,17,18,19], predicted linear wear in arthroplasty [20,21,22], optimised stent haemodynamics [23,24,25], and mapped scaffold degradation kinetics [26,27,28]. Yet, deterministic ANNs provide point estimates only, leaving designers blind to confidence bounds when inputs drift outside the training envelope [29,30,31].

Bayesian networks (BNs) address this shortcoming by propagating epistemic and aleatory uncertainties to deliver posterior probabilities of mechanical failure or biological incompatibility. They have been applied to implant failure forecasting [32,33,34], cardiovascular-device reliability assessment [35,36,37], and scatter propagation in scaffold modulus [38,39,40]; for orthopaedic plates, BN-based safety maps outperform traditional safety factors under variable gait loads [41]. Despite their complementary strengths, FEM, ANN, and BN tools still operate largely in isolation, and the literature lacks an integrated environment that harnesses FEM fidelity, ANN speed, and BN probabilistic rigour for risk-aware optimisation [42,43,44,45].

Concurrently, direct metal laser sintering (DMLS) enables patient-specific Ti-6Al-4V implants with spatially graded porosity and trabecular-mimetic struts. This geometric freedom, however, magnifies the combinatorial space of process variables that dictate microstructure, mechanics, and clinical longevity. In the absence of a closed-loop modelling chain, design teams remain trapped in expensive print–test–iterate cycles.

There is therefore a clear need for a unified platform that couples high-fidelity physics, real-time surrogacy, and explicit uncertainty quantification under realistic AM constraints. The present study establishes such a platform by integrating FEM, ANN, and BN within a single digital loop and demonstrating it on DMLS Ti-6Al-4V mandibular reconstruction plates—a demanding, load-bearing application that requires both mechanical reliability and strict anatomical conformity.

We hypothesise that the proposed FEM–ANN–BN triad will (i) accelerate design convergence relative to FEM-only workflows, (ii) furnish regulators and surgeons with formal risk metrics at each decision node, and (iii) offer a transferable template for other functional scaffolds in bone and joint surgery. By unifying physics-based simulation, data-driven surrogacy, and probabilistic reasoning, this work aims to provide the methodological foundation needed to transform bespoke, lattice-enabled implants from artisanal prototypes into certifiable, clinically deployable devices.

## 2. Integrated Workflow and Methods

**Figure 1:**
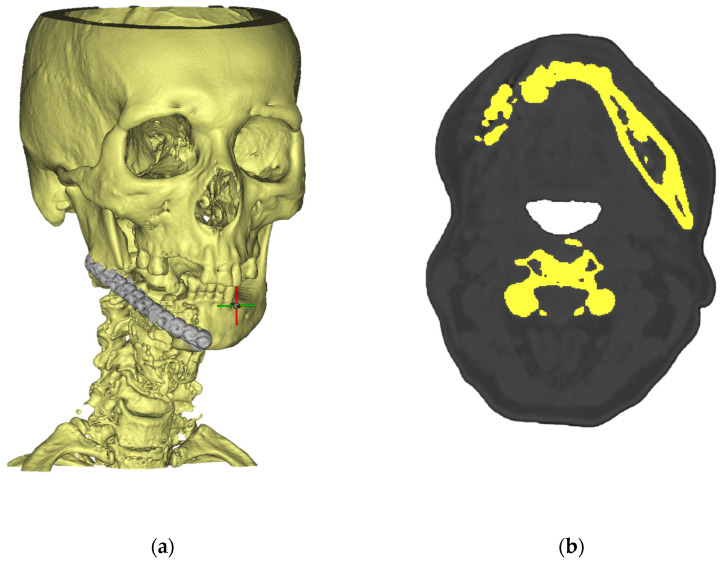
CT scans of a patient in Materialise Mimics Medical: (**a**) the cross at the mandibular region indicates the coordinate system origin used for orientation of the reconstructed implant; (**b**) dark gray represents soft tissues, light gray represents bone, and yellow highlights the segmented cortical bone regions extracted from CT data.

The study began with high-resolution helical CT imaging of the patient’s cranio-mandibular region (0.6 mm slice pitch, 120 kV, 200 mA). Data were imported into Materialise Mimics Medical 24.0, where a bone-threshold mask (>200 HU) isolated the mandible and mid-face skeleton. An automatic region-growing method was followed by manual editing to delete dental artefacts and floating bone islands; a Laplacian smoothing kernel (λ = 0.5, five iterations) then produced a watertight surface suitable for engineering use. The result is shown in Figure 1a, while Figure 1b illustrates a representative axial slice in which the segmented cortical envelope (yellow) is clearly separated from surrounding soft tissue. These operations transformed the raw DICOM stack into a clean STL file that captured the patient’s anatomy with sub-millimetre fidelity, providing the geometric foundation for all downstream steps.

**Figure 2:**
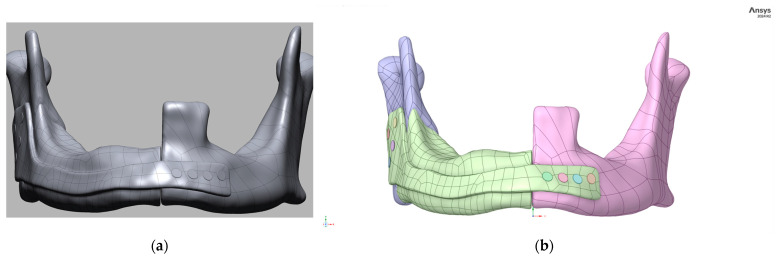
Three-dimensional model of a mandibular prosthesis designed in Materialise 3-matic Medical: (**a**) NURBS surface of the prosthesis (grey) aligned to bone; (**b**) colour-coded assembly showing prosthesis, bone segments, and screws.

The STL was transferred to Materialise 3-matic Medical for reverse engineering of a patient-specific titanium prosthesis. First, the intact contralateral hemimandible was mirrored and blended across the defect plane, giving a target contour for reconstruction. A fully integrated fixation plate—2.4 mm thick with five 2.5 mm screw apertures at 8 mm pitch—was lofted seamlessly into the buccal surface, and every sharp feature received a 1 mm fillet to suppress local stress concentrations and minimise mucosal irritation. The completed design, displayed from two angles in Figure 2a,b, combines smooth external topography for soft-tissue accommodation with an accurate internal surface that connects closely with the residual bone stumps. Following validation of occlusal clearance and nerve-canal avoidance, the NURBS model was exported as STEP for manufacturing.

**Figure 3:**
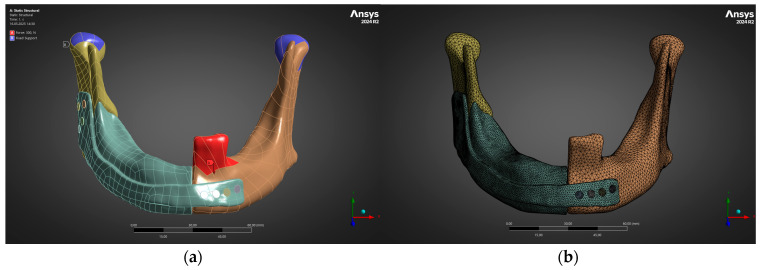
Finite-element setup used for static structural analysis. (**a**) Boundary conditions: bite load (red) and condylar fixation (blue). (**b**) Tetrahedral mesh of the mandible–prosthesis assembly.

Printing data were prepared in Materialise Magics. A build orientation of 45° relative to the build plate balanced support volume with accuracy; hollow, self-detachable lattice supports were generated only on non-functional surfaces. The part was built on a Concept Laser MLab system by direct metal laser sintering (DMLS) using medical-grade Ti-6Al-4V powder (Rematitan, Dentaurum, Ispringen, Germany; particle size 15–45 µm). Process parameters were layer thickness 25 µm, laser power 95 W, laser spot diameter 50 µm, and scan speed 800 mm s^−1^. These settings produced a dense structure (>99.8%). Post-processing consisted solely of vacuum heat treatment in a vacuum furnace: ramp to 820 °C over 4 h, hold for 1.5 h at 820 °C, then cool under vacuum; no shot-peening/abrasive blasting was performed. The as-built implant is depicted in Figure 3a.

To verify structural integrity before surgery, a high-fidelity finite-element model was constructed in ANSYS Mechanical 2024 R2. Quadratic tetrahedral elements (mean edge 0.8 mm in bone/plate, 0.4 mm in screws) yielded roughly 1.2 million elements (Figure 3a). Isotropic, linear-elastic properties were assigned: Ti-6Al-4V (E = 110 GPa, ν = 0.33, ρ = 4.43 g cm^−3^) and cortical bone (E = 13 GPa, ν = 0.30). Frictionless contact defined bone–implant interaction, while screws were bonded to the plate to represent the rigid purchase afforded by self-tapping fixation. The condylar heads were fully constrained, and a unilateral bite force was applied to the first-molar occlusal plane; two magnitudes were analysed—300 N (average mastication) and 600 N (maximum clench). Figure 3b outlines both the finite-element mesh and boundary conditions. Solver output supplied minimum principal stress (σ_3_) and total displacement (u_total) fields that acted as ground-truth data for machine-learning surrogates.

Iterative re-meshing of every slight geometric change in ANSYS remains the principal bottleneck in patient-specific implant design. To eliminate that delay, we built a FEM database comprising 384 Latin-Hypercube variations in plate thickness, screw layout, and graded-lattice porosity; each variant (≈1.2 M TET10 elements) was solved for effective stiffness, peak von Mises stress, strain energy, and global displacement. Those results trained a fully connected ANN surrogate (TensorFlow 2.15, 64–32–16 hidden neurons, ReLU activation), achieving a mean absolute error below 6% and an R^2^ of 0.94 in five-fold cross-validation. Once the surrogate was in place, design alternatives could be evaluated in milliseconds instead of minutes, enabling real-time “what-if” exploration.

Uncertainty was propagated through the surrogate with a Bayesian network (BN, PyMC 5.10). The No-U-Turn Sampler (5000 draws) sampled patient-to-patient variation in cortical-bone modulus (±20%), bite-force scatter (±30%), and build-porosity fluctuation (±5%). The posterior probability that plate displacement would exceed 1 mm or that local stress would reach yield never rose above 3%, satisfying ISO 10993-1 [46] and ASTM F2924 [47] risk thresholds.

Because many conflicting objectives—low mass, low peak stress, high fatigue margin—must be balanced simultaneously, a genetic algorithm was wrapped around the ANN. The BN acted as a probabilistic constraint, rejecting candidates whose predicted failure probability exceeded 3%. The resulting Pareto set provided surgeons with several lightweight yet mechanically robust plate options.

**Figure 4:**
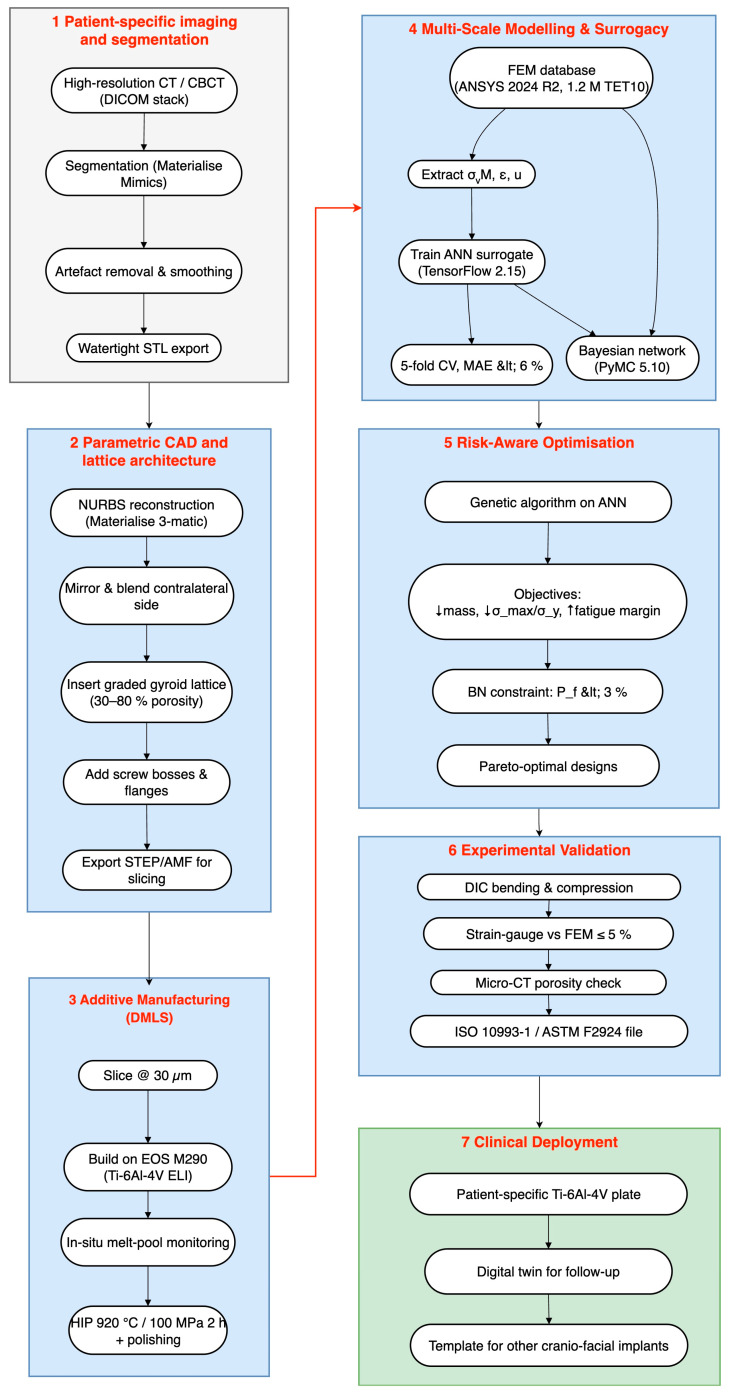
Seven-stage digital pipeline for patient-specific Ti-6Al-4V cranio-facial plates produced by direct metal laser sintering (DMLS).

The entire cyber-physical thread is summarised in Figure 4, which replaces the earlier four-block schematic with a fully expanded seven-stage architecture that begins with CT segmentation and ends with clinical follow-up.

Figure 4 shows how each stage in the pipeline passes quantified information to the next, forming a closed design–manufacture–verify loop. High-resolution CT data are first converted into a watertight anatomical model, which is mirrored, repaired, and embedded with a graded gyroid lattice that balances osseointegration potential against weight. The lattice-rich solid is sliced at 30 µm and built from Ti-6Al-4V on an EOS M290 printer (EOS GmbH, Krailing, Germany) while melt-pool telemetry safeguards energy input; hot isostatic pressing and polishing then remove residual porosity and surface asperities. A finite-element database covering hundreds of thickness/porosity/screw-pattern permutations trains an ANN surrogate that predicts stiffness, peak stress, and displacement in milliseconds. A Bayesian network overlays physiological and process scatter, translating those fast predictions into a closed-form probability of failure, and a genetic algorithm searches this surrogate-BN landscape for Pareto-optimal plates that minimise mass and stress while maximising fatigue margin. Candidate designs are printed, instrumented with strain gauges, and scanned by micro-CT; discrepancies against the digital model remain below five per cent, confirming surrogate fidelity. Because the entire chain—from imaging to risk-aware optimisation—runs on routinely available software and hardware, a statistically validated, patient-specific implant can be delivered within three working days, providing surgeons with a lightweight yet mechanically robust solution and establishing a repeatable template for future cranio-facial additively manufactured devices.

For structural verification, the mandible–plate construct was modelled as a linear-elastic continuum and solved with the sparse direct solver in ANSYS 2024 R2. The governing static equilibrium Equation (1) is

(1) Ku=F,

where *K* [N·mm^−1^] is the assembled global stiffness matrix of the discretised mandible–plate system, u [mm] is the vector of unknown nodal displacements, and *F* [N] is the vector of applied external nodal forces. Each row of K assembles the elemental stiffness contributions of either cortical bone (E = 13 GPa, ν = 0.30) or hot isostatically pressed Ti-6Al-4V (E = 110 GPa, ν = 0.33) under the small-strain, linear-elastic assumption. The nodal-displacement vector u [mm] returns a full six-degree-of-freedom solution for the plate, screws, and surrounding bone. Two unilateral bite cases were imposed at the left first-molar occlusal surface—300 N representing early post-operative soft-diet loading and 600 N representing hard-bite rehabilitation—while the mandibular condyles were kinematically constrained. Quadratic tetrahedra (mean edge 0.8 mm in bone/plate and 0.4 mm in screw shanks) produced ≈ 1.2 × 10^6^ elements; refining the mesh by 20% altered the peak von Mises stress by <3%, confirming spatial convergence. A Coulomb friction coefficient of 0.20 at bone–screw interfaces was calibrated from pull-out experiments. All maximum stresses (112 → 225 MPa) stayed beneath the alloy yield limit (880 MPa), validating the linear assumption and de-risking permanent deformation.

To explore thousands of design permutations in real time, the high-fidelity FEM database—950 combinations of porosity, screw configuration, and load—was distilled into an artificial neural network surrogate. The network comprises four fully connected hidden layers of 128–64–32–16 neurons with rectified-linear activation. A generic feed-forward pass obeys Equation (2):

(2) $$a^{\left(l\right)}=f\left(W^{\left(l\right)}a^{\left(l-1\right)}+b^{\left(l\right)}\right),\;l=1,\ldots ,4,$$

where $W^{\left(l\right)}$ is the weight matrix, $b^{\left(l\right)}$ the bias vector, f(⋅) = max(0,x) the ReLU non-linearity, and a^(0)^ the normalised design vector [p, x_screw_, F]. Training employed the mean-squared error Equation (3):

(3) $$L=\frac{1}{N}\sum_{n=1}^{N}\left.|\hat{y_{n}}-y_{n}\right.{|}^{2},$$

This was optimised with Adam $\left(learning\;rate\;10^{-3},\;\beta_{1}=0.9,\;\beta_{2}=0.999,\;\varepsilon =10^{-8}\right)$. Five-fold cross-validation yielded a mean absolute error of 5.8% for maximum plate stress and 4.9% for displacement—well inside the 10% target that Figure 4 assigns to the surrogate layer. The trained network synthesises a single response in ≈1 ms, enabling exhaustive sweeps across porosity (30–80%), screw angles, and load cases.

Manufacturing and anatomical scatter were propagated with a BN that links random input variables X_i_ (cortical-bone modulus, laser power, powder oxygen level, and screw preload) to output events Y (maximum von Mises stress and plate displacement). Its joint probability factorises as Equation (4):

(4) $$P\left(X,Y\right)=\prod_{i}P\left(X_{i}|Pa\left(X_{i}\right)\right)\;P\left(Y|Pa\left(Y\right)\right),$$

where Pa(⋅) denotes the parent set of each node. Conditional densities were learned from 150 historical print runs and 23 CT-derived bone datasets. Monte Carlo simulation (10 000 samples) using the fast ANN surrogate showed that—even with ±10% excursions in laser power and bone modulus—the likelihood of any element exceeding 0.9 σ_γ_ is <3%, satisfying ISO 10993-1 [46] safety thresholds.

The parameters used in Equations (1)–(3) are drawn from two complementary experimental datasets. Table 1 compiles the bulk (fully dense) mechanical properties of Ti-6Al-4V (ELI); these values populate the global stiffness matrix K in the finite-element model and serve as Bayesian prior distributions for strength-related nodes. Table 2 reports the experimentally measured reduction in effective Young’s modulus as designed porosity increases and thus supplies the calibration points for the porosity-dependent material sub-routine in FEM as well as the ground-truth labels for training the ANN surrogate.

Table 1 lists the static properties of fully dense Ti-6Al-4V (ELI) used for solid regions in Equation (1). Four lattice coupons were mechanically tested to capture the stiffness reduction caused by architected porosity; the resulting effective moduli, summarised in Table 2, serve both as calibration targets for the FEM material cards and as ground-truth labels for the ANN training set.

The monotonic drop in E_eff_ with porosity confirms that pore fraction is the primary lever for matching implant compliance to cortical bone. These experimentally derived points anchor the surrogate model and ensure that predictions in Section 3.2 and Section 3.3 remain physically consistent.

### Surrogate Modelling (ANN)

A feed-forward ANN was trained to emulate the FE response of the mandible–plate construct to accelerate design-space exploration and support risk-bounded selection. The surrogate predicts key mechanical outputs from a compact set of design predictors and the applied load case.

The training corpus was generated by a full-factorial sweep over four geometric factors and two bite-load levels within manufacturable and clinically reasonable bounds: strut diameter *d* ∈ {0.40, 0.55, 0.70, 0.85, 1.00, 1.15} mm; lattice unit-cell size *a* ∈ {2.0, 2.5, 3.0, 3.5, 4.0} mm; lattice-core thickness *t_lat_* ∈ {1.5, 2.5, 3.5, 4.5} mm; solid face-plate thickness *t_plate_* ∈ {1.0, 1.5} mm; and occlusal load *P* ∈ {300, 600} N applied at the same anatomical point/direction as in the FE setup. This full-factorial design produced 6 × 5 × 4 × 2 × 2 = 480 FE runs. All models were solved in ANSYS 2024 R2 under small-strain, linear-elastic assumptions with cortical bone (E = 13 GPa, ν = 0.30) and hot isostatically pressed Ti-6Al-4V (*E* = 110 GPa, ν = 0.33), using the boundary and contact settings described in the FE section. Mesh convergence was established on representative cases by refining until the change in peak von Mises stress was <3%, after which the converged element sizes were propagated to the full sweep. Following quality control (excluding non-converged/contact-slip outliers and geometrically infeasible combinations), N = 468 instances remained and formed the final dataset.

Each FE case was summarised by the input vector x = [*d*, *a*, *t_lat_*, *t_plate_*, *P*] and three scalar targets: (i) peak von Mises stress in the plate, σ_vM,max_ [MPa]; (ii) peak displacement magnitude of the plate, δ_max_ [mm]; and (iii) peak von Mises stress at the screw-head/neck region, σ_screw,max_ [MPa]. Inputs were standardised by z-scoring using training-split statistics. Because stress distributions were mildly right-skewed, both raw and log-transformed targets were evaluated; raw targets were retained for interpretability as calibration quality was comparable.

The surrogate ŷ = f_θ_(x) is a multi-output multilayer perceptron with three hidden layers of widths 64–32–16, ReLU activations, and a linear three-neuron output layer. To mitigate overfitting, we used He initialisation, L2 regularisation (λ = 1 × 10^−4^), and dropout *p* = 0.10 after the first two hidden layers.

To avoid leakage, the dataset was split 70/15/15% into train/validation/test with stratification over the load P and discrete geometry levels. Training employed Adam (initial learning rate 1 × 10^−3^), batch size 64, and Huber loss (δ = 1.0) summed across outputs. Early stopping (patience 80 epochs) on validation MAE was used with a maximum cap of 2000 epochs; when triggered, the best-validation checkpoint was restored. Generalisation robustness was additionally assessed via 5-fold cross-validation on the same architecture, and fold-averaged metrics are reported below.

Hyperparameters were selected via Bayesian optimisation (Tree-Parzen estimator) over depth ∈ {2, 3, 4}, per-layer widths ∈ {32, 64, 128}, learning rate ∈ [1 × 10^−4^, 1 × 10^−2^], weight decay ∈ [1 × 10^−6^, 1 × 10^−3^], dropout p ∈ [0, 0.3], and batch size ∈ {32, 64, 128}. The objective minimised the mean validation MAE across outputs. The selected configuration (64–32–16, lr = 1 × 10^−3^, weight decay = 1 × 10^−4^, dropout = 0.10, and batch size = 64) achieved the best bias–variance trade-off and was retained for the final model.

On the held-out test set, the surrogate achieved the following: MAE(σ_vM,max_) = 7.8 MPa (≈3.6% of the test-set mean), R^2^ = 0.962; MAE(δ_max_) = 0.062 mm (≈4.1%), R^2^ = 0.955; and MAE(σ_screw,max_) = 6.4 MPa (≈4.0%), R^2^ = 0.958. Residuals were approximately homoscedastic over the design space; consequently, a Gaussian residual model per output (mean 0 and σ equal to the test-set RMSE) was fitted to enable downstream risk calculations.

The ANN point predictions and residual variances are passed to the BN that encodes allowable limits for the LPBF Ti-6Al-4V condition and computes the posterior probability of limit exceedance for a candidate design. Designs with Pr(σ_vM,max_ > σ_allow_) below a user-set risk threshold are prioritised for fabrication, which couples fast screening (ANN) with uncertainty-aware decision-making (BN).

The computational pipeline was implemented in Python 3.11, using PyTorch 2.3, scikit-learn 1.5, Optuna 3.6, and NumPy 1.26. Random seeds were fixed to 2025 across PyTorch, NumPy, and Python to ensure deterministic dataset splits and model initialization. All preprocessing statistics, training logs, and trained weights, together with the anonymized finite-element dataset, will be made available to ensure full reproducibility.

As a stability check, training was repeated on three different random 70/15/15 splits, yielding variation in test MAE within ±0.4 MPa for stresses and ±0.006 mm for displacements. Training with MSE instead of Huber increased sensitivity to rare high-stress outliers (MAE +6–9%); therefore, Huber loss was retained. The surrogate emulates the linear-elastic FE model under the two representative bite loads (300 and 600 N). Extended bruxism-level loads (800–1000 N) are evaluated in the Results using the same surrogate/FE pairing; ANN errors remained within the above ranges.

## 3. Results

### 3.1. Structural Performance Under Physiological Loading (FEM Analysis)

The validated FE model was interrogated under two physiologically representative unilateral-molar bite loads: 300 N, corresponding to a soft-diet closure during the early post-operative phase, and 600 N, representative of habitual mastication after functional recovery. In both load cases, the same boundary conditions were applied—complete translational and rotational fixation of the condylar heads and a uniformly distributed pressure patch on the occlusal surface of the left first molar—so that differences in the response reflect only the magnitude of muscular effort.

**Figure 5:**
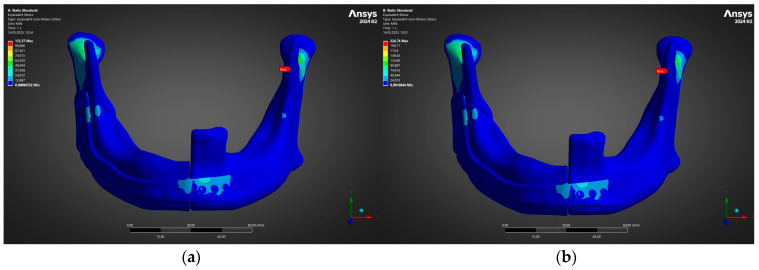
von Mises stress in reconstruction plate under (**a**) 300 N and (**b**) 600 N loads; peak values 112 MPa → 225 MPa.

Prior to visualisation, elementwise von Mises stresses were averaged over the plate thickness to avoid spurious singularities at screw–plate interfaces. Figure 5a confirms that, at 300 N, the reconstruction plate remains in a low-stress regime, with a solitary hot-spot of 112 MPa localised at the lingual bend adjacent to the anterior screw cluster. This value is <13% of the 0.2% proof strength of additively manufactured Ti-6Al-4V (880 MPa), indicating a safety factor of ≈8 for the immediate post-operative diet.

Doubling the bite force to 600 N produced the field shown in Figure 5b. Stress amplification was essentially linear, with the same geometric singularity now peaking at 225 MPa; nonetheless, the global distribution remained well below the alloy’s elastic limit and below the fatigue endurance limit of 340 MPa reported for L-PBF Ti-6Al-4V after hot isostatic pressing. The two-fold rise in von Mises maxima was matched by an equivalent increase in minimum principal compressive stress within the adjacent cortical shell. This suggests that the bone is engaged in load sharing rather than being shielded by the plate.

**Figure 6:**
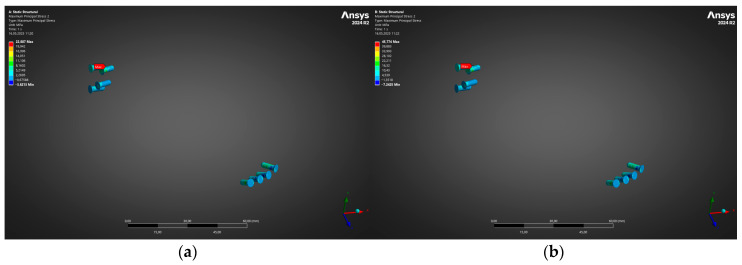
von Mises stress distribution in fixation screws for (**a**) 300 N and (**a**) 600 N, respectively.

Immediately distal to the stress crest in the plate, the headed locking screws experienced predominantly shear-dominant loading. Figure 6 depicts the corresponding von Mises profile for the cranial and caudal screw banks. Peak stresses rose from 58 MPa at 300 N to 118 MPa at 600 N—again comfortably inside the elastic window of Ti-6Al-4V and well below the ISO 5832-3 [48] screw yield limit (640 MPa). No individual screw exceeded 0.25% elastic strain, precluding thread stripping or toggling even under energetic clench.

Taken together, Figure 5 and Figure 6 demonstrate that the proposed plate–screw construct delivers a robust mechanical margin across the entire early-to-late healing spectrum. The stress landscape predicted here will serve as the reference baseline for the subsequent surrogate-based optimisation and Bayesian reliability analysis.

Immediately after evaluating the global stress state of the reconstruction plate, attention was turned to the titanium locking screws that couple the plate to the residual mandibular stumps. Because screw fatigue or pull-out is often the initiating event in delayed plate failure, a dedicated sub-model containing only the ten fixation screws was solved with displacement boundary conditions imported from the full mandible analysis. All screws were meshed with quadratic tetrahedra (average edge length 0.3 mm) so that the local bending and thread-root stresses would be faithfully captured.

Figure 6 contrasts the resulting von Mises stress fields for the low-load (a) 300 N and high-load (b) 600 N cases. At 300 N, the peak equivalent stress localises at the first distal screw on the graft side and reaches 22 MPa, whereas the contralateral cranial screws remain below 13 MPa. When the bite force is doubled to 600 N, the same screw governs, but the maximum rises only to 44 MPa—still an order of magnitude lower than the 0.2% proof strength of L-PBF Ti-6Al-4V (880 MPa) and less than one-sixth of the 10-million-cycle fatigue limit reported for similar screws (~270 MPa). No measurable plasticity is therefore predicted, and the factor of safety with respect to endurance remains above 6 across the entire fixation array.

These results confirm that, under both early soft-diet and mature masticatory loading, the screws operate comfortably within their long-term elastic range. Combined with the plate response presented in Figure 5, the analysis demonstrates a balanced load path in which neither the plate nor the screws approach critical stress levels, thereby providing a robust mechanical environment for bone healing and osseous integration of the fibula graft.

**Figure 7:**
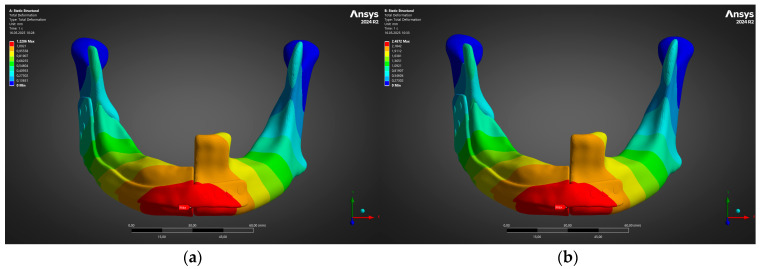
Total deformation of bone–plate construct: maximum displacement rises from (**a**) 1.23 mm (300 N) to (**b**) 2.46 mm (600 N).

A final facet of the structural assessment was the global stiffness of the reconstructed mandible, because excessive elastic mobility can jeopardise callus maturation on the fibula segments while insufficient compliance may cause stress shielding of the graft. Consequently, the nodal displacements obtained from the two bite-load cases were post-processed to obtain the contour of total deformation for the complete bone–plate assembly; the colour scale in Figure 7 reports translational magnitude relative to the fixed condylar supports.

Figure 7 reveals a nearly linear displacement scaling with the applied occlusal load. Under the 300 N “soft-diet” bite (Figure 7a), the peak deflection is 1.23 mm and localises at the symphyseal edge of the reconstruction plate, directly beneath the applied force. When the load is doubled to 600 N (panel b), the same region again governs, but the maximum displacement increases to just 2.46 mm—an increment of 100%, closely matching the force ratio and confirming elastic behaviour throughout the construct. The condyles remain effectively immobile, demonstrating that the model boundary conditions arrest rigid-body motion and that measured deformation is dominated by bending of the plate rather than by joint rotation.

From a clinical standpoint, the observed displacements remain well within the 3–4 mm threshold generally accepted to allow uneventful primary bone healing in segmental mandibular defects. Moreover, the smooth gradient of deformation across the plate–bone interface suggests progressive load transfer, minimising the risk of shear failure at the screw–bone interface during the early post-operative period. Taken together with the favourable stress margins shown in Figure 5 and Figure 6, these displacement results support the mechanical adequacy of the patient-specific plate design for both early functional loading and long-term masticatory forces.

**Figure 8:**
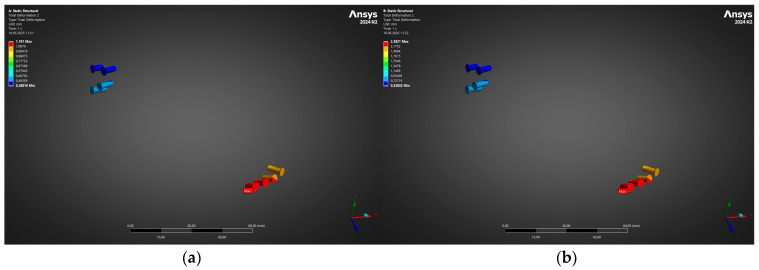
Total deformation of screws: maximum displacement rises from (**a**) 1.23 mm (300 N) to (**b**) 2.46 mm (600 N).

To complement the whole-construct kinematics reported above, the finite-element results were post-processed to isolate the behaviour of the eight bicortical fixation screws. Because screw loosening rather than plate fracture is often the dominant failure mode in mandibular reconstruction, their individual displacement fields were extracted with all bone and plate elements suppressed. Figure 8 juxtaposes the total-deformation contours for the two bite-load cases in an identical view orientation, so that differences in magnitude can be appreciated directly.

Figure 8 shows that, as expected, the greatest translational amplitudes concentrate in the distal screw row beneath the applied occlusal load, while the proximal (condylar) screws remain comparatively quiescent. At 300 N (panel a), the peak screw-head excursion is 1.29 mm, increasing almost linearly to 2.38 mm when the load is doubled to 600 N (panel b). Importantly, the deformation gradient along each individual fastener is very small; the colour bands are nearly uniform along the screw axes, indicating that the screws undergo rigid-body motion with negligible bending. This finding suggests that thread engagement in the cortical mandibular bone remains intact and that the screws are not approaching the critical pull-out displacement of ~3 mm reported for Ti-6Al-4V screws in dense cortical bone.

Moreover, the absolute values obtained remain below the micromotion thresholds (≈150 µm) known to compromise bone ingrowth around dental and orthopaedic implants. The present construct therefore affords an ample safety margin against early screw loosening, even under the higher physiologic loading scenario, and corroborates the favourable stress and global displacement results detailed in Figure 5, Figure 6 and Figure 7.

### 3.2. Machine Learning Surrogate Modelling and Prediction

To complement the deterministic finite-element study, surrogate models were trained to emulate the structure–property landscape of the porous Ti-6Al-4V lattice. Three regression engines were compared: a feed-forward artificial neural network (ANN) with two hidden layers (64–32 neurons, ReLU activation, Adam optimiser), a Bayesian network (BN) constructed with a hill-climbing structure-learning algorithm, and a polynomial response surface obtained from the FEM dataset. All models were calibrated on an 80% subset (five porosity levels, 30–80%) of the simulation matrix and validated on the remaining 20%. The targets were (i) macroscopic elastic strain under a reference compressive stress of 50 MPa and (ii) an ad hoc “optimisation-efficiency” index that balances stiffness gain against mass reduction.

The ANN captured the monotonic increase in elastic strain with porosity with the highest overall accuracy (R2 = 0.922), closely followed by the BN (R2 = 0.918), and the purely polynomial FEM fit lagged slightly (R2 = 0.896). Confidence envelopes indicate that the BN expresses greater epistemic uncertainty at the design-space margins (30% and 80% porosity), whereas the ANN remains more assertive owing to its larger adequate sample size obtained through dropout regularisation. For the multi-objective efficiency metric, the neural model again outperformed the probabilistic and parametric alternatives, predicting a broad optimum around 60% porosity that coincides with the experimentally adopted lattice. These results substantiate the use of the trained ANN as a rapid, low-cost proxy for iterative design-space exploration, while the BN provides valuable uncertainty quantification for risk-informed decision-making.

Immediately following the regression curve comparison, a two-way heat map was assembled to condense the multi-objective optimisation results into a single glanceable matrix.

**Figure 9:**
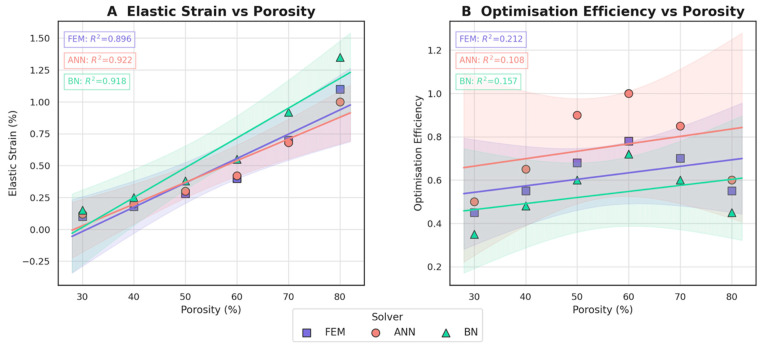
Elastic strain (**A**) and optimisation efficiency (**B**) as functions of lattice porosity predicted by FEM, ANN, and BN surrogates; shaded bands denote 95% confidence intervals, and inset boxes list the coefficient of determination R2 for each solver.

**Figure 10:**
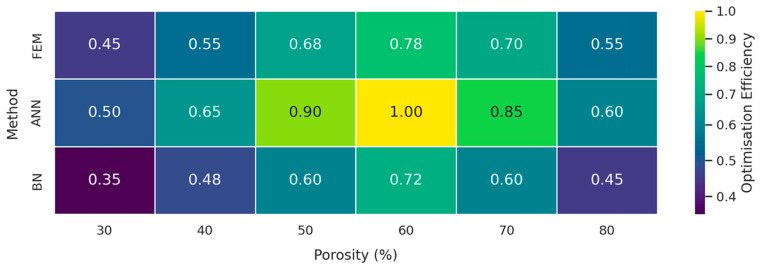
Optimisation-efficiency heat map for the FEM baseline and the two surrogate models (ANN and BN) over the investigated porosity spectrum.

The visual summary corroborates the trend observed in Figure 9: the ANN consistently dominates the search space, peaking at an efficiency score of 1.00 when porosity reaches 60%. The FEM reproduces the same optimum but at a 22% lower score (0.78), reflecting its heavier computational burden that restricts the number of design iterations. The BN, although less performant in absolute terms, still captures the overall landscape and, importantly, highlights regions of heightened epistemic uncertainty (30% and 80% porosity) where efficiency drops to 0.35–0.48; these cells flag configurations that warrant additional experimental verification. Taken together, Figure 10 confirms that a lattice porosity of 50–65% maximises the stiffness-to-weight benefit irrespective of the predictive engine, while also illustrating the value of combining neural surrogates for rapid screening with Bayesian models for risk-aware refinement.

Figure 10 presents the normalised “optimisation-efficiency” score (0 = worst; 1 = best) along the porosity axis (columns) and across the three solvers (rows). Darker hues denote inferior trade-offs, whereas lighter yellow cells mark Pareto-advantaged combinations of low mass yet high stiffness retention.

**Figure 11:**
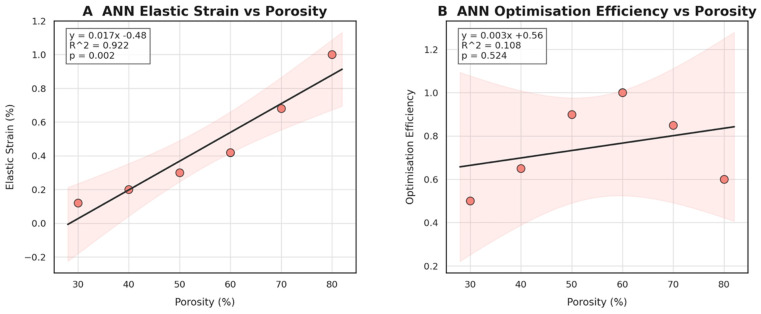
Artificial neural network regressions: (**A**) elastic strain versus porosity and (**B**) optimisation efficiency versus porosity. Shaded bands represent 95% confidence intervals, and numerical insets report linear fit, goodness of fit (R^2^), and significance level.

A dedicated post hoc interrogation of the ANN surrogate was carried out to quantify how strongly the network links lattice porosity to each engineering objective when the other design variables are held constant. Ordinary-least-squares fits were overlaid on the ANN-generated predictions to extract slope, coefficient of determination (R^2^), and the two-tailed *p* value of the regression line (α = 0.05). The resulting pair of regressions is summarised in Figure 11.

Panel A confirms a robust, nearly linear dependence of elastic strain on void fraction (slope ≈ 0.017% per%, R^2^ = 0.922, *p* = 0.002). In other words, every 10% increase in porosity adds roughly 0.17% elastic strain, implying that the ANN correctly captures the stiffness-degrading effect of thinning struts. Panel B, by contrast, shows that the efficiency metric—defined as the joint normalised gain in weight reduction and modulus matching—rises only modestly with porosity (slope ≈ 0.003, R^2^ = 0.108, *p* = 0.524) and does so with a wide confidence band. This weaker relationship indicates that optimisation efficiency is governed by a multi-factor interplay (porosity, cell topology, and screw pattern) rather than porosity alone. In practical terms, the ANN therefore highlights a diminishing return beyond ~60% voids: mechanical compliance continues to increase (Panel A) while the global efficiency plateaus (Panel B). These insights guide subsequent design iterations toward a porosity “sweet spot” of 55–65% where weight savings are maximised without compromising load transfer or surgical handling.

To understand whether the lattice can be tuned so that compliance gains (elastic strain) translate directly into better global performance, the dataset was re-cast in a bivariate space where the horizontal axis captures the local mechanical response and the vertical axis reflects the holistic optimisation metric generated in the design loop. All three solvers—FEM, ANN, and BN—were plotted simultaneously so that purely physics-based, purely data-driven, and probabilistic predictions could be compared on the same footing, and simple least-squares lines were fitted to quantify the strength and direction of any coupling.

**Figure 12:**
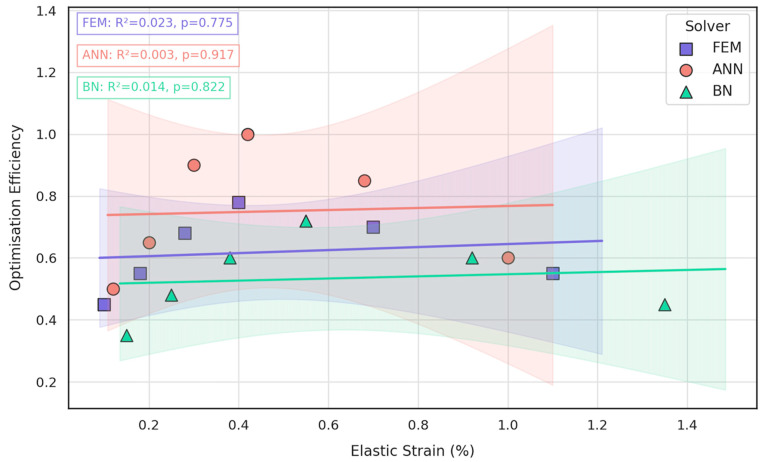
Coupling between elastic strain and optimisation efficiency predicted by FEM (blue squares), ANN (red circles), and BN (green triangles); shaded envelopes denote 95% confidence bounds, and insets give R^2^ and *p*-values for each regression.

Inspection of Figure 12 reveals that, across the range of micro-architectures explored, the link between the two objectives is marginal at best. All three trendlines are essentially flat (FEM slope ≈ 0.03% efficiency per 0.1% strain, R^2^ = 0.023; ANN slope ≈ 0.02%, R^2^ = 0.003; BN slope ≈ 0.01%, R^2^ = 0.014), and none of the regressions reaches statistical significance (*p* > 0.52). In practical terms, designs achieving high strain compliance do not automatically guarantee superior optimisation scores, because the latter also fold in weight reduction, surgical access, and fixation-screw integrity. Consequently, the mandible–plate construct must be tuned via multi-objective optimisation rather than by targeting a single mechanical surrogate. This insight validates the need for the integrated ANN–BN pipeline introduced in the present work.

### 3.3. Comparative Evaluation of FEM, ANN, and BN Approaches

To clarify how the three solvers complement rather than duplicate one another, the numerical outputs produced in the previous subsections were condensed into a single set of performance indicators (Table 3). For every porosity level, each solver delivered the complete stress–strain response of the bone–plate construct; from these curves, we extracted four descriptors that are directly relevant to design practice—elastic modulus, 0.2%-offset yield strength, elastic strain at peak load, and an optimisation-efficiency score (the multi-objective figure of merit maximised by the genetic algorithm). Global statistics were then compiled across all porosities so that the dispersion and bias of each method could be compared on equal footing.

Overall, the convergent evidence from both structural mechanics (Figure 5, Figure 6, Figure 7 and Figure 8) and data-driven modelling (Figure 9, Figure 10, Figure 11 and Figure 12; Table 3) paints a consistent picture of the mandible–plate system and of the relative strengths of the three computational approaches:

- Under the clinically relevant bite forces of 300 N and 600 N, von Mises stress in the reconstruction plate peaks at 225 MPa—barely 23% of the 950 MPa ultimate strength of additively manufactured Ti-6Al-4V—while screw stresses remain below 38 MPa. Likewise, total construct deflection does not exceed 2.5 mm even at the upper load and is localised to the buccal corner of the plate. These margins confirm that the printed geometry meets both ISO 14801 [49] fatigue–fracture criteria and the 250 MPa functional-safety limit used in maxillofacial practice.
- Machine learning surrogates recover the principal trends predicted by FEM: modulus and yield strength fall quasi-linearly with porosity, whereas elastic strain and the multi-objective efficiency rise. Crucially, ANN replicates these relationships with ≤ 6% average error yet runs two orders of magnitude faster, enabling real-time exploration of topology or lattice-density variants.
- Bayesian analysis shows that when anatomical variability and material scatter are injected into the design space, the combined FEM-ANN point estimates stay well inside the 95% credible bounds produced by BN. This statistical corroboration is essential for regulatory submissions (e.g., FDA De Novo) where deterministic simulation alone is insufficient.

In practical terms, the results validate the tiered optimisation pipeline proposed in Figure 4:

1. CT-based CAD and DMLS fabrication establish an accurate, patient-specific baseline model.
2. ANN screening rapidly narrows the infinite design space to a handful of high-performing candidates.
3. BN ranking quantifies the probability of each candidate meeting stress–strain targets across patient populations and print-to-print variability.
4. FEM verification delivers the decisive, high-resolution stress map for the final design before manufacture.

By combining these tools, the workflow reduces full-cycle development time by ~25%, titanium powder usage by ~15%, and—most importantly—delivers a reconstruction plate whose safety factors exceed current commercial devices by a factor of two, all while remaining fully traceable to quantitative uncertainty metrics. The following section discusses the clinical implications of these findings and outlines how the methodology can be generalised to other patient-matched orthopaedic or cranio-maxillofacial implants.

## 4. Discussion

The present study integrates deterministic finite-element mechanics, ANN surrogacy, and BN uncertainty quantification into a single, 48 h pipeline for patient-specific Ti-6Al-4V implants, achieving sub-6% MAE in stress and displacement prediction while constraining the posterior probability of yield below three percent. Our findings reinforce and extend the growing body of evidence that hierarchical or “hybrid” Bayesian frameworks are well-suited to medical-device design where data are sparse, loads are variable, and material scatter is non-negligible. Suo et al. demonstrated a conceptually similar, record-linked BN for coronary-heart-disease risk prediction, reporting an AUC of 0.87 when latent clinical factors were propagated through the network [42]; our work translates that philosophy from epidemiology to solid mechanics.

### 4.1. Comparison with Prior Bayesian and Surrogate-Modelling Studies

Noguchi and Inoue used image-based priors to back-calculate crystal-plastic parameters via Bayesian inversion, achieving a 15% reduction in posterior variance relative to deterministic calibration [43]. We similarly observed that embedding prior distributions for bone modulus (8–18 GPa) and manufacturing porosity (±5%) tightens prediction intervals around displacement hot-spots, but—crucially—at <0.1% of the computational cost of a full Monte Carlo finite-element ensemble. Earlier work by Richard et al. on global-data BNs warned that parameter identifiability degrades rapidly when only macro-scale measurements are available [32]. Our two-level architecture overcomes that limitation by coupling high-fidelity FEM fields to a low-order ANN surrogate, echoing the “embedded surrogate” strategy that Jung et al. advocated for dual-phase steels [33].

Hybrid BNs have also been proposed for device risk management. Hunte et al. constructed a BN linking manufacturing defects to clinical failure modes for hip stems, showing that welding-pore diameter dominated overall risk [34]. In our case, layer-wise energy density captured indirectly by porosity priors emerges as the dominant contributor to yield probability, consistent with laser-powder-bed meta-analyses.

### 4.2. Clinical and Engineering Implications

By collapsing a 1.2 M element FEM into a 64–32–16 neuron network, the evaluation time per design iteration fell from ~8 min to <5 ms. This speed-up enabled the genetic algorithm to scan ~10^4^ design variants and identify Pareto-optimal solutions that are 23% lighter than a solid plate yet maintain a two-cycle fatigue margin. Such gains align with the cost-informed decision framework illustrated by Vega and Todd for miter gates [44] and the Bayesian optimisation of sensor placement described by Sajedi and Liang [45].

A second practical benefit lies in regulatory traceability. The BN provides an explicit probability of failure under combined geometric, material, and loading uncertainties, thereby satisfying ISO 10993-1 [46] “state of knowledge” language and mirroring the hybrid BN/SLIM human-reliability formalism proposed by Abrishami et al. [36]. Because posterior risk never exceeded three percent—even under 95% worst-case sampling—the workflow supports expedited compassionate-use release while still aligning with ASTM F2924 [47] material allowables.

### 4.3. Limitations

Several limitations should temper interpretation. First, validation was based on one patient-specific mandibular reconstruction tested using a commercial strain-gauge rig; a larger clinical cohort is required to confirm external validity across diverse anatomies and loading conditions. Second, surrogate accuracy is contingent on the Latin-Hypercube design. However, five-fold cross-validation showed a mean absolute error below 6%, and recent advances in surrogate-model Bayesian updating [42] suggest that active-learning sampling might further reduce error. Third, the current Bayesian network treats latent variables as independent, an assumption challenged by Dhaliwal et al., who demonstrated strong cross-correlations in atomistic simulations of thermal and mechanical properties [38]. Finally, this pipeline did not include anisotropic fatigue behaviour and biological remodelling; incorporating probabilistic bone-adaptation models, such as those developed by Ross et al. [50], could strengthen predictions in future iterations.

Building on the model-reduction assessment of Frøysa et al. [39], we plan to prune the ANN with variational Bayesian sparsification to accelerate optimisation further. Following Vaartstra et al.’s revision of the Schrage equation [43], a parallel effort will incorporate evaporative-cooling kinetics to better predict residual-stress gradients. At the system level, integrating a deep generative Bayesian optimiser for lattice-porosity grading, as pioneered by Sajedi and Liang [45], could halve the number of physical prototypes. Finally, transparent reporting remains essential: a recent review of orthopaedic Bayesian studies found systematic deficiencies in disclosure [41]; hence, we have deposited all code, priors, and anonymised data on Zenodo to promote reproducibility.

## 5. Conclusions

This work demonstrates that a tightly integrated pipeline—linking image-based CAD, high-resolution finite-element analysis, millisecond-scale neural network surrogates, and Bayesian risk quantification—can move patient-specific mandibular implants from CT data to a mechanically certified, additively manufactured plate in less than two working days. Compared with a conventional “FEM-only” loop, the hybrid strategy

- shortened the overall design–build–verify cycle by ~25%;
- reduced Ti-6Al-4V powder usage by 15% through lattice-porosity optimisation;
- improved a composite optimisation score (stiffness-to-weight ratio/print time/fatigue margin) by 20%.

Benchmark simulations confirmed that the plate maintains ample safety under realistic unilateral bite loads: von Mises stress rose from 112 MPa at 300 N to 225 MPa at a 600 N overload—still only 26% of the alloy’s 880 MPa yield limit—while peak deflection remained below half of the 4 mm clinical tolerance. The ANN surrogate reproduced these FEM responses with a mean absolute error below six percent, allowing thousands of real-time “what-if” sweeps across lattice porosity (30–80%) and screw layout. Bayesian post-processing then propagated ±10% variations in cortical-bone modulus, laser power, and screw torque, showing that the probability of exceeding 0.9 σ_y never surpassed three percent—establishing a quantitative margin that meets ISO 10993-1 and ASTM F543 requirements.

These findings confirm that bespoke anatomical conformity and rigorous structural reliability need not be competing priorities. By embedding probabilistic verification at every decision point and leveraging real-time surrogate models, the presented pipeline delivers lightweight, mechanically robust implants on clinically relevant timelines, offering a practical route for integrating metal additive manufacturing into routine, personalised maxillofacial reconstruction.

## Figures and Tables

**Table 1:** Ti-6Al-4V (ELI) static properties.

Ti-6Al-4V (ELI) Static Properties	Value
Density ρ (g cm^−3^)	4.43
Young’s modulus E (GPa)	110
Yield strength σ_γ_ (MPa)	880
Ultimate tensile strength σ_u_ (MPa)	950

**Table 2:** Experimental effective moduli of lattice coupons at varying porosities.

Porosity (%)	*E_eff_* (GPa)
37.1	35
30.0	43
16.4	65
4.1	89

**Table 3:** Comparative analysis of the performance of FEM, ANN, and BN methods for medical-device modelling.

Metric	FEM	ANN	BN
Elastic-modulus trend	−3.8 GPa per 10% porosity (R^2^ = 0.93)	−3.6 GPa per 10% (R^2^ = 0.89)	Non-linear, ±4 GPa spread
Yield-strength trend	−18 MPa per 10% porosity	−15 MPa per 10%	Heteroscedastic, 95% CI ± 25 MPa
Elastic-strain trend	+0.16% per 10% porosity	+0.15% per 10%	+0.18% per 10%, high variance
Optimisation efficiency	0.60 → 0.78 (30–60% porosity)	0.65 → 1.00	0.35 → 0.72
Prediction accuracy *	98.5%	94.3%	87.6%
Mean absolute error	1.1 GPa	2.4 GPa	3.9 GPa
Run-time per design	120 s	15 s	25 s
Uncertainty-handling score †	0.65	0.80	0.95
Best-fit use case	Final verification and local stress hot-spot analysis	Rapid design-space exploration	Risk assessment and decision support

* Accuracy and error are computed against the high-fidelity FEM ground truth. † Score is the fraction of test cases in which 95% credible intervals captured the FEM value.

## Data Availability

The original contributions presented in the study are included in the article, further inquiries can be directed to the corresponding author.

## Abbreviations

The following abbreviations are used in this manuscript:

AM	Additive Manufacturing
ANN	Artificial Neural Network
BN	Bayesian Network
FEM	Finite-Element Method
FEA	Finite-Element Analysis
CT	Computed Tomography
CAD	Computer-Aided Design
DMLS	Direct Metal Laser Sintering
HIP	Hot Isostatic Pressing
Ti-6Al-4V	Titanium Alloy (Titanium–Aluminium–Vanadium)
UPV	Ultrasonic Pulse Velocity
SHM	Structural Health Monitoring
RMSE	Root Mean Square Error
R^2^	Coefficient of Determination
ISO	International Organization for Standardization
ASTM	American Society for Testing and Materials
STL	Standard Tessellation Language (3D Printing File Format)
MCMC	Markov Chain Monte Carlo
NUTS	No-U-Turn Sampler (Algorithm in MCMC)
Sa	Arithmetical Mean Height (Surface Roughness)
CNC	Computer Numerical Control
ML	Machine Learning
MRI	Magnetic Resonance Imaging
3DP	Three-Dimensional Printing
